# Enhanced carbon dioxide conversion at ambient conditions via a pore enrichment effect

**DOI:** 10.1038/s41467-020-18154-9

**Published:** 2020-09-08

**Authors:** Wei Zhou, Qi-Wen Deng, Guo-Qing Ren, Lei Sun, Li Yang, Yi-Meng Li, Dong Zhai, Yi-Hong Zhou, Wei-Qiao Deng

**Affiliations:** 1 Institute of Molecular Sciences and Engineering, Institute of Frontier and Interdisciplinary Science, Shandong University, Qingdao, 266237 P. R. China; 2 Collage of Hydraulic & Environmental Engineering, China Three Gorges University, Yichang, Hubei 443002 P. R. China; 3 State Key Laboratory of Molecular Reaction Dynamics, Dalian National Laboratory for Clean Energy, Dalian Institute of Chemical Physics, Chinese Academy of Sciences, Dalian, 116023 P. R. China; 4 University of the Chinese Academy of Sciences, Beijing, 100039 P. R. China

**Keywords:** Catalyst synthesis, Heterogeneous catalysis, Molecular dynamics

## Abstract

Chemical fixation of carbon dioxide (CO_2_) may be a pathway to retard the current trend of rapid global warming. However, the current economic cost of chemical fixation remains high because the chemical fixation of CO_2_ usually requires high temperature or high pressure. The rational design of an efficient catalyst that works at ambient conditions might substantially reduce the economic cost of fixation. Here, we report the rational design of covalent organic frameworks (COFs) as efficient CO_2_ fixation catalysts under ambient conditions based on the finding of “pore enrichment”, which is concluded by a detailed investigation of the 10994 COFs. The best predicted COF, Zn-Salen-COF-SDU113, is synthesized, and its efficient catalytic performance for CO_2_ cycloaddition to terminal epoxide is confirmed with a yield of 98.2% and turnover number (TON) of 3068.9 under ambient conditions, which is comparable to the reported leading catalysts. Moreover, this COF achieves the cycloaddition of CO_2_ to 2,3-epoxybutane under ambient conditions among all porous materials. This work provides a strategy for designing porous catalysts in the economic fixation of carbon dioxide.

## Abstract (web summary)

Currently the cost of CO_2_ chemical fixation remains high because of harsh reaction conditions. Here, the authors report a covalent organic framework screened from 10994 candidates as the efficient CO_2_ fixation catalyst under ambient conditions based on the finding of a “pore enrichment effect”.

## Introduction

As an important component of the air, CO_2_ is well known as a greenhouse gas. With the continuous consumption of fossil fuels, the CO_2_ concentration in the atmosphere reached 410 ppm in 2017^1^, which was the highest value in human history, and the concentration continues to increase. The resulting greenhouse effect has started to affect the stability of global ecosystems, leading to the frequent occurrence of global extreme weather. Currently, there are two industrialized technologies to reduce CO_2_ emissions, capture or conversion, which already have been working at annual ten-million-ton scale. However, either capture or conversion technology imposes a high cost because of the facilities requiring the high temperature or pressure or both. The global economy cannot afford billion-ton scale CO_2_ emission reduction. Therefore, a method to capture and convert CO_2_ at ambient conditions is a potential solution for economic CO_2_ emission reduction.

In 2013, our previous work found a Salen-conjugated microporous polymer (CMP) can capture and convert CO_2_ into valuable chemicals at ambient conditions^2^. As far as we know, metal-Salen catalysts can realize the CO_2_ transformation under relatively mild conditions^3–7^. Moreover, the pore structure in CMP can further lower the requirements of reaction temperature and pressure. With the goal to improve the reaction conditions, extensive efforts have been dedicated by combining different moieties possessing high catalytic activity and the porous polymer and framework materials, such as porous organic polymers (POPs)^8^, microporous organic polymer (MOP)^9^ and metal-organic frameworks (MOFs)^10–14^. Besides, the metal-free ionic polymers have also been applied in the syntheses of porous catalytic materials under the mild conditions^15,16^. All these researches indicate that the incorporation of catalytic active moieties into the porous materials is a feasible way to realize the fixation CO_2_ under ambient conditions by achieving capture and conversion of CO_2_ simultaneously. The CMPs, POPs, MOPs, MOFs, etc. are actually library compounds with millions of possible candidates. There is no doubt that a better candidate can be found by the large-scaled screening method, which has been used in various purposes in literatures^17–25^.

In this work, we use large-scale computation screening method to address the best candidate in a constructed library of COFs and demonstrate the best predicted COF. First, we build a virtual library, including 10994 COF structures using Salen-metal (M-Salen) as the catalytic centre. The CO_2_ adsorption performances at 298 K and 1 bar are theoretically investigated. Based on the simulation results, we analyse the CO_2_ distribution characteristic within the frameworks of the materials with the best and worst CO_2_ adsorption performance. Second, we find a pore enrichment effect by observing the enriched local concentration of CO_2_ inside special pore structures. With this pore enrichment effect, the reaction rate can be significantly increased, leading to the efficient catalytic process. Finally, we synthesize the best predicted COF structure (abbreviated as Zn-Salen-COF-SDU113) and measure its catalytic performance for CO_2_ molecule fixation. The obtained COF exhibits excellent CO_2_ conversion performance under atmospheric pressure and room temperature. The Zn-Salen-COF-SDU113 catalyst shows high catalytic activity and excellent stability and recyclability towards terminal epoxides (PO) and internal epoxides (2,3-epoxybutane) under ambient conditions.

## Results

### Simulation prediction

**Fig. 1:**
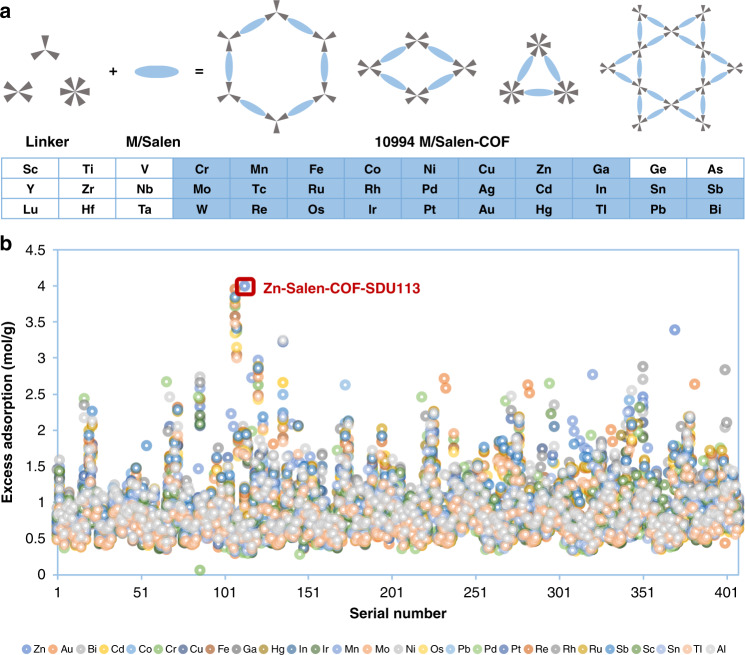
Topology diagram and excess CO_2_ adsorption amount. **a** The polygon skeletons and the metal element types for the 10994 kinds of Metal/Salen-COFs. Three types of linker groups and Metal/Salen build four topology structure, which from left to right are hexagonal, single-pore rhombic, trigonal and dual-pore kagome, respectively. The chemistry structures for all the linker groups were listed in the Supplementary Information. Twenty-eight elements were chosen as the central metal of metal/salen. **b** The simulated excess adsorption amount (mol g^−1^) of CO_2_ in 10994 kinds of Metal/Salen-COFs at 298 K and under 1 bar.

**Fig. 2:**
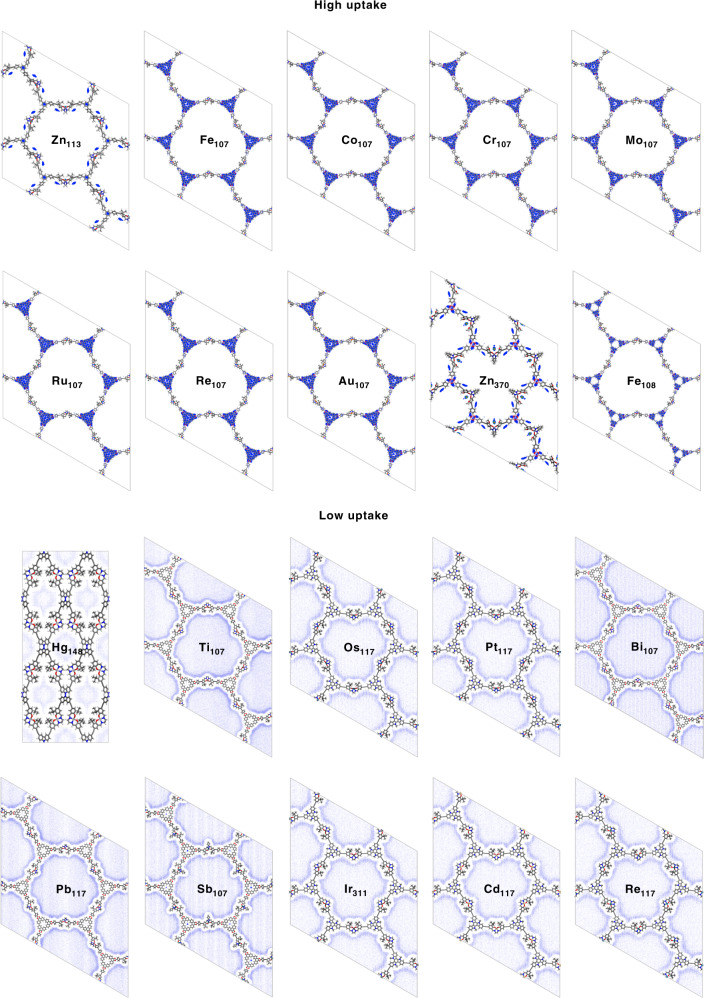
CO_2_ average densities. Ten Metal/Salen-COFs with the highest excess CO_2_ adsorption amount (upper) and ten Metal/Salen-COFs with the lowest excess CO_2_ adsorption amount (lower). The different shades of colour distinguish the degree of CO_2_ uptake. Dark blue represents a large average density.

To construct a structure library of Salen-COFs, we choose three types of linker groups to build different topological structures, as shown in Fig. 1. Then, twenty-seven metal elements are used as the metal centre of M-Salen, and the alkane substituent groups usually present in the Salen molecule are also considered in the construction process of the Salen-COFs. Finally, 10994 M-Salen-COF structures were built for the structure dataset. The excess adsorption amounts of CO_2_ by the 10994 M-Salen-COF structures, which were optimized by molecular dynamic methods, were simulated. Most of the excess value is distributed in the range from 0.5 to 1.5 mol g^−1^ and the excess amounts for 252 structures are higher than those of Co-CMP (1.802 mol g^−1^), which can capture and convert CO_2_ under ambient conditions^2^. For all COFs, the maximum value of 3.99 mol g^−1^ is for Zn-Salen-COF-SDU113. It is worth noting that the second-highest value (3.94 mol g^−1^) belongs to the No.117 COF structure with the Fe-Salen compound. Moreover, the other six highest excess amounts are all for COFs with the No. 117 COF structure. The metal elements in the M-Salen compound are Co, Cr, Mo, Ru, Re and Au, which show excess amounts of 3.83, 3.82, 3.75, 3.72, 3.58 and 3.47 mol g^−1^, respectively. According to the analyses of the CO_2_ average density shown in Fig. 2, the captured CO_2_ molecules in these seven COFs with the same COF structure are mainly concentrated between the two linker groups along the direction of the 2D pore. This type of linker group, which is a large conjugated structure, provides the capture environment for CO_2_. The result of adsorption between two layers is that the introduction of the corresponding metal atom promotes the structural deformation of the No.117 COF. Although the structure leads to CO_2_ enrichment, only a small portion of the captured CO_2_ molecules can directly interact with the M-Salen compound in these COFs. Similarly, Fe_108_ exhibits the same distribution feature. In the constant Zn_113_ (Zn-Salen-COF-SDU113) and Zn_370_, some of the captured CO_2_ molecules gather around the Zn-Salen fragment, which is the catalytic reaction centre in the chemical fixation of CO_2_. For these M-Salen-COFs with high CO_2_ uptakes, we found a pore enrichment effect. Precisely due to the enrichment of CO_2_ molecules, the excess adsorption amount is improved significantly. This gathered phenomenon in the pore may change the reaction environment of the chemical fixation of CO_2_.

To further state the pore enrichment effect, we also analysed the average densities of CO_2_ for the COFs with the worst adsorption performance, as shown in Fig. 2. The ten COFs exhibit the low excess adsorption amounts of 0.277, 0.297, 0.306, 0.309, 0.312, 0.315, 0.328, 0.330, 0.334 and 0.339 mol g^−1^ for Hg_148_, Tl_107_, Os_117_, Pt_117_, Bi_107_, Pb_117_, Sb_107_, Ir_311_, Cd_117_ and Re_117_/Salen-COFs, respectively. The results of the CO_2_ average densities clearly indicate that the captured CO_2_ molecules are evenly distributed around the COFs. In these COFs, there is no strong enough molecular interaction to effectively capture the CO_2_ molecules. Interestingly, three Salen-COFs (Tl_107_, Bi_107_ and Sb_107_) with the No. 117 COF are among the ten worst COFs. In these three COFs, only a few CO_2_ molecules were distributed between the linker groups along the direction of the 2D pore, and CO_2_ enrichment is very weak. The measurement of the distance between the two linker layers for Au_107_ and Tl_107_ shows that the interlayer spacing decreases from 7.29 to 5.36 Å, thus blocking CO_2_ enrichment between the two adjacent conjugated groups. The above analysis on the ten M-Salen-COF structures with the highest excess amount shows that a “pore enrichment effect” obviously enhances the CO_2_ adsorption performance. But, no enrichment is not found in the low CO_2_ uptake structures. The difference indicates that the pore enrichment is benefit to the CO_2_ storage in the porous materials.

**Fig. 3:**
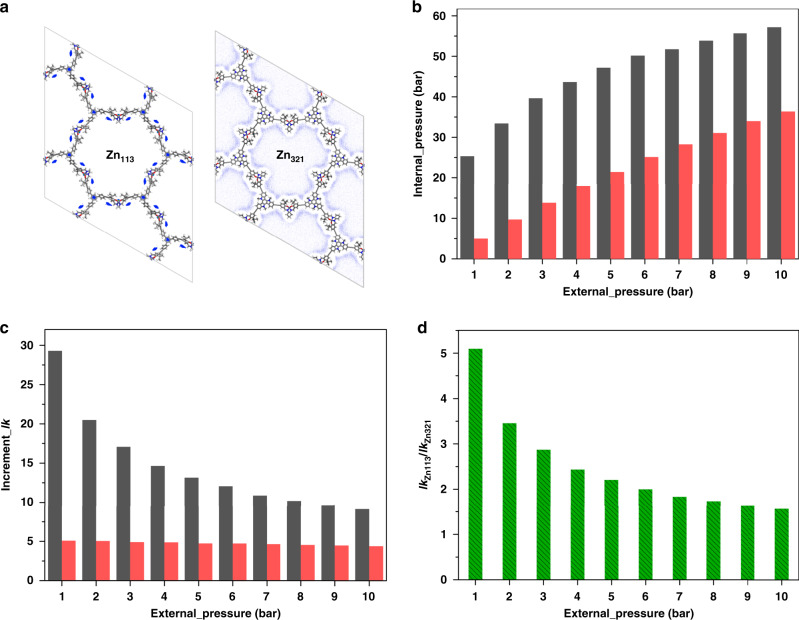
Pore enrichment effect on the internal pressure and reaction rate. **a** CO_2_ average densities of in Zn-Salen-COF-SDU113 and 321. **b** The variation trend of internal pressure along with increasing external pressure. **c** The increment of the reaction rate (*Ik*) for Zn-Salen-COF-SDU113 (*Ik*_Zn113_) and 321 (*Ik*_Zn321_) under internal and external pressure, respectively. **d** The ratio of *Ik*_Zn113_/*Ik*_Zn321_. In **b**, **c**, the black and red colours represent Zn-Salen-COF-SDU113 and 321, respectively.

Besides the influence on the uptake amount, we further study the pore enrichment effect on the catalytic reaction. The external and internal pressures of the gas-filled Zn_113_ and Zn_321_ were compared. In Zn_113_, there is obvious CO_2_ enrichment, but the captured CO_2_ in Zn_321_ is evenly distributed around the framework. Figure 3b shows that the internal pressure increases with the external pressure. When the external pressure is 1 bar, the internal pressure for Zn_113_ and Zn_321_ is 25.3 and 4.97 bar, respectively. As the external pressure increases to 10 bar, the internal pressure reaches 57.2 and 36.4 bar, respectively. The gap between Zn_113_ and Zn_321_ indicates that the pore enrichment effect favours an increase in the gas pressure in the pore. We further studied the pore enrichment effect on the reaction rate (*k*_PC_) of the coupling reaction of CO_2_ and propylene oxide (PO) into propylene carbonate (PC), based on the rate Eq. (1) as follow,

1 $$k_{{\mathrm{PC}}} = Aexp\left( {\frac{{ - E_{\mathrm{a}}}}{{RT}}} \right)\left[ {{\mathrm{CO}}_2} \right]^m\left[ {{\mathrm{PO}}} \right]^n$$

where, *A* is the pre-exponential factor, *E*_a_ is the activation energy, *R* is the gas constant, *T* is the temperature, [CO_2_] and [PO] is the concentration of reactants, and *m* and *n* is the reaction order. Here, they both equal to 1. Additionally, because the central metal elements of the catalysts are both zinc and the species of reactants are the same, we assumed that *E*_a_ and *A* in the catalytic processes using Zn_113_ and Zn_321_ are the same, thus the reaction rate can be approximately only proportional to [CO_2_] inside the pore. We compared the increment values (*Ik*) of *k*_Zn113_ and *k*_Zn321_ under internal pressure to the ones under external pressure, respectively, as shown in Fig. 3c, d. As a whole, when the external pressure increases gradually, the pore enrichment effect will weaken. However, at 1 bar external pressure, *Ik*_Zn113_ is the highest (29.3 times) and *Ik*_Zn321_ is only 5.07. As the external pressure increases, *Ik*_Zn113_ and *Ik*_Zn321_ fall to 9.13 and 4.39, respectively. This result shows that the increment change of *k*_Zn113_ is more obvious, especially at low pressure. But for Zn_321_, the increment changes a little from 1 to 10 bar. It reflects that the pore enrichment effect in Zn_113_ is much stronger than Zn_321_ as shown in the analysis results of CO_2_ average densities. Due to the obvious pore enrichment effect, it is easy for Zn_113_ to implement catalytic reactions requiring high-pressure conditions under atmospheric pressure.

It is worth nothing that the pore enrichment effect is different from the confinement effect which mainly acts on the electronic structure of the reactants or products, thus changes the energy barrier of the catalytic processes. And yet, the pore enrichment effect mostly caused by the behaviour of numerous molecules in the pore. We will expound the differences in the later ‘Discussion’ section.

### Experiment verification

**Fig. 4:**
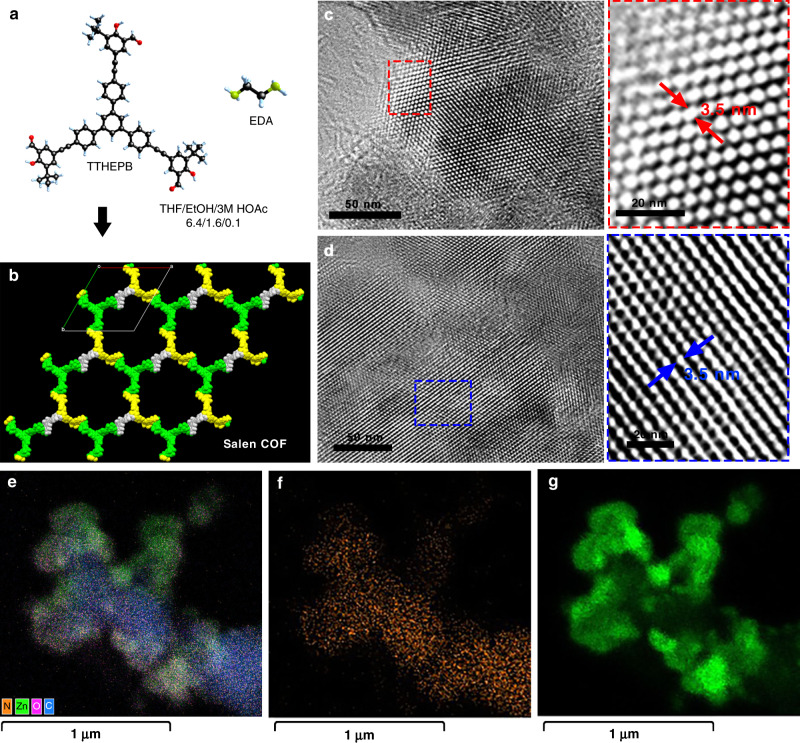
Synthesis and structure of Zn-Salen-COF-SDU113. **a**, **b** Synthesis of Zn-Salen-COF-SDU113. **c**, **d** TEM images of Zn-Salen-COF-SDU113 (the dotted border of different colours corresponds to different areas). **e**–**g** Energy-dispersive X-ray spectroscopy (EDS) mapping images of Zn-Salen-COF-SDU113 (orange: N elements; green: Zn elements).

Based on the simulated results above, the corresponding experiments have been carried out to verify the pore enrichment effect. We synthesized the best Salen-COF, Zn-Salen-COF-SDU113, by using the similar synthesis method^2,26–28^. Both Fourier transform infrared (FT-IR, Supplementary Figs. 1 and 2) and soild state ^13^C nuclear magnetic resonance (^13^C NMR, Supplementary Fig. 3) measurement results indicate the formation of imine bonds via Schiff condensation of the ligand TTHEPB and EDA^20,21^. The experimental Powder X-ray diffraction (PXRD, Supplementary Fig. S4) pattern matched well with the simulated pattern constructed from the AA stacking of the 2D layers (Fig. 4b). The unit cell parameters were also obtained (*a* = *b* = 43.887 Å, *c* = 8.418 Å, *α* = *β* = 90°, *γ* = 120°, *R*_p_ = 6.98% and *R*_wp_ = 4.95%, for more details, see Supplementary Table 2). As shown in Supplementary Fig. 5, scanning electron microscopy (SEM) images revealed that all Zn-Salen-COF-SDU113 powders retained a spherical morphology with an average particle size of 0.5–2 μm. It is worth noting that the transmission electron microscopy (TEM) images exhibit a regular channel structure (Fig. 4c); those images exhibit a uniform honeycomb structure, which is in good agreement with the predicted structure. The measured average diameter of those channels is ~3.5 nm, which also matches the distance between two adjacent pore walls (incident electron beam perpendicular to the channels, Fig. 4c). The pore structure was explored by nitrogen adsorption and desorption isotherms at 77 K and CO_2_ adsorption analyses at 298 K (Supplementary Figs. 7 and 8). The nitrogen adsorption isotherms revealed uptake below *P*/*P*_0_ = 0.05. The Zn-Salen-COF-SDU113 samples have a BET surface area of 1143 m^2^ g^−1^. When *P*/*P*_0_ approaches 0.0167, the CO_2_ uptake reaches 19.8 mg g^−1^. According to the nonlocal density functional theory, the pore size distribution of Zn-Salen-COF-SDU113 is 3.5 nm, indicating that it is a mesoporous material. Moreover, the pore size distribution is in good agreement with the pore diameter optimized space-filling model and the average diameter measured from the HRTEM images also match the simulative pore diameter and pore size distribution. We notice that part of the channel structure is distorted, this fact suggests structural changes that are probably caused by the activation process and high vacuum and high energy electron beam conditions during HRTEM testing^29^. Based on the above results, Zn-Salen-COF-SDU113 with **hcb** topology was successfully synthesized. By increasing the dosage of organic ligands by 20 times, we found that the synthesized Zn-Salen-COF-SDU113 can still keep good crystallinity. As shown in Fig. 4e–g, the EDS mapping proved the homogeneous distribution of Zn^2+^ ions in the crystal structures. Moreover, the ICP analysis shows that the Zn element content is 4.18 wt%.

**Fig. 5:**
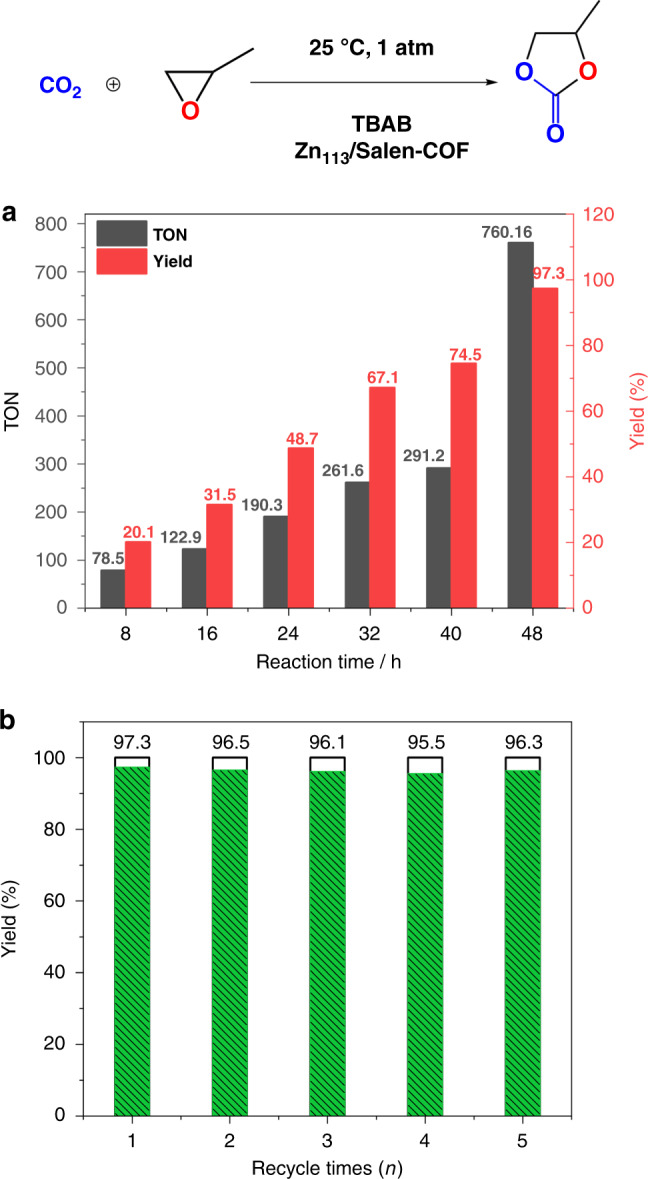
Catalytic performance for Zn-Salen-COF-SDU113. **a** The yield of PC depending on the reaction time and **b** recycling stability of Zn-Salen-COF-SDU113 at atmospheric pressure and room temperature.

Zn-Salen-COF-SDU113 demonstrates excellent activity and selectivity for catalysing the reaction of CO_2_ and terminal epoxides (PO) into PC under ambient conditions. After metalation with Zn^2+^, the obtained Zn-Salen and Zn-Salen-COF-SDU113 powders were mixed with the nBu_4_NBr co-catalyst (TBAB), and then, the reaction system was purged three times with a CO_2_ atmosphere. This reaction was performed in pressure tubing at 1 bar and 298 K. As shown in Supplementary Table 3, Entries 1–5 show the catalytic performances of different catalysts under the same experimental conditions. The test results indicate that the co-catalyst TBAB plays a key role in catalytic conversion of CO_2_ and PO. The coupling reaction between CO_2_ and PO catalysed by Zn-Salen-COF-SDU113 yields 10.3% PC without the co-catalyst TBAB. However, the co-catalyst TBAB showed a yield of 20.4% in our previous work^1^. When mixed with TBAB, Zn-Salen-COF-SDU113 can effectively accelerate the catalytic reaction with an improved yield of 98.2% and a turnover number (TON) value of 3068.9, which are much higher than those of Zn-Salen tested under the same conditions (Yield: 79.2%, TON: 162.3). Fig. 5a shows the relationships between the yield and TON (PC) and reaction time at atmospheric pressure and room temperature. It is obvious that both the yield and TON values are proportional to the reaction time. To further confirm that our simulation studies are appropriate and useful in guiding the COF design, another COF (Zn-Salen-COF-SDU11) with lower expected performance to Zn-Salen-COF-SDU113 were synthesized for comparison. As is consistent with simulation studies, under the same reaction conditions, Zn-Salen-COF-SDU11 shows catalytic activity with a yield of 84.2% and a turnover number (TON) value of 1142.7, which are much lower than Zn-Salen-COF-SDU113 (Yield: 98.2%, TON: 3068.9). The trend of theoretical calculation is consistent with that of actual catalytic measurement. Moreover, upon further exploring the recycling stability of Zn-Salen-COF-SDU113, it is obvious that there is no significant decrease in the yield after 5 cycles of the CO_2_/PO coupling reaction. Moreover, the obtained Zn-Salen-COF-SDU113 still maintains good crystallinity after five cycles (for more details, see the PXRD patterns in Supplementary Fig. 4), indicating the excellent stability and recyclability of the solid COF catalyst during the catalytic CO_2_/PO reaction. Moreover, we also test the catalytic activity towards internal epoxides (2,3-epoxybutane) with a substantial yield of 74.4% and a TON value of 533.9 at ambient conditions, which have never been achieved under ambient conditions by other porous materials including CMPs, POPs, MOPs and MOFs.

## Discussion

For our designed M-Salen-COFs, the embedded catalytic moieties retain the catalytic activity for CO_2,_ and the porosity of the framework provides an environment space for gas capture. Variety of viable porous materials can be used based on this concept^29–33^. It is worth noting that the embedded strategy is not only the superposition of two functions. The chemical reactions are also affected by the pore environment in the porous materials, such as the confinement effect. The confinement effect indicates that the electronic orbitals and reaction barrier of the pore structure will change when the molecules enter into the pores of zeolite, affecting chemical reactions^34–36^. It was found that the confinement effect can be synergistically cooperate with nano effect to catalysis reactions^27,37–39^. The loaded nanoparticles inside carbon nanotubes change the catalytic performance of the nanoparticles. The analysis results show that the relatively narrow pores significantly increased the catalytic activity. In recent years, people have extended this concept. It is believed that the reactions in porous materials with proper pore size, including zeolites, MOFs, COFs and other molecular cage materials, can be all affected by the pore environment and change the electronic orbital or charge distribution of the reactive molecules to change the reaction barrier^40–42^. In general, the electronic confinement more likely to happen when the size of the adsorbate molecule is similar to the size of the pore^34^. With the narrow or crowded pores relative to the molecule size, the molecular structures of the adsorbate are more easily affected by the confined environment, so the confinement effect is the dominant factor. When the pore size increases or the adsorbate size decreases, we believe that the confinement effect should gradually weaken because the adsorption space is large enough for the adsorbate under the circumstances^43^, and the pore enrichment effect caused by the molecular aggregation turn into the most important factor. For the CO_2_ adsorption in the M-Salen-COFs designed by us, the enrichment just changes the concentration of reactants around the catalytic centre, not the obviously electronic structure of reactant. It is an effect of changing the equilibrium conditions of the catalytic reaction. Therefore, the pore enrichment will promote the thermodynamic process for the small-size molecule catalysed by using relatively large-pore porous materials. We infer that the reactions involving with gases is the most common chemical reaction. Therefore, we think that the pore enrichment effect can be widely applied for the small-size gas molecules, hydrogen, oxygen and ethylene so on.

In summary, we built about ten thousand of M-Salen-COF structures using Salen-metals as the catalytic centre, and their corresponding CO_2_ capture performances were theoretically investigated by studying the CO_2_ distribution, CO_2_ adsorption abilities and molecular aggregation. According to the simulated results, we synthesized the corresponding COF materials (Zn-Salen-COF-SDU11, Zn-Salen-COF-SDU113 and Zn-Salen-COF-SDU128), and it exhibited outstanding catalytic PO/CO_2_ coupling reaction activity and excellent stability and recyclability. Moreover, Zn-Salen-COF-SDU113 can even transform internal epoxides (2,3-epoxybutane) under ambient conditions. Based on the theoretical and experimental results, we find the pore enrichment effect to explain the effect on the CO_2_ fixation process caused by the molecular aggregation state. This work provides an effective strategy, by using pore enrichment effect, to achieve excellent catalysts with high catalytic activity, chemical stability and recycling ability by combining simulations and experiments. This strategy provides a direction for the oriented design of COFs and other porous materials for CO_2_ economic fixation.

## Methods

### Simulation methods

To obtain stable frameworks, we optimize the geometries for each COF through annealing method. The classical molecular dynamics package LAMMPS^43^ is used with the universal force field (UFF)^44^. A periodic unit cell is employed. In simulated annealing, the structures are first heated to and equilibrated at 500 K for 1 ns. They are then cooled to 300 K at a rate of 25 K/100 ps. The last step is equilibration at 300 K for 1 ns. Based on the dynamic trajectories of equilibration, we select the lowest-energy geometry as the starting geometry in the next annealing process. After repeated cycling, the optimization is completed when the energy convergence threshold reaches 1 × 10^−4^ kcal/mol. The NVT ensemble is used during annealing, with a Nose–Hoover thermostat for temperature and volume control. To determine the CO_2_ adsorption capacity, the grand canonical Monte Carlo (GCMC) method was used with Dreiding force field. To obtain an accurate measure of molecule loading, we specify 1,000,000 equilibration steps before the production stages and 3,000,000 Monte Carlo steps in the production stage of the simulation. Charges in the GCMC simulations are set through the QEq charge equilibration method. Excess uptake is defined as the number of CO_2_ molecules that can be adsorbed around the pore wall of porous materials compared to the free volume^45,46^.

### Synthesis and characterization methods

The Salen-COF powder were synthesized by traditional solvothermal method. In typically, 50 mg TTHEPB were dissolved in a mixture of 6.4 ml THF and 1.6 ml EtOH. Then, 30 μL EDA was added, the mixed solution quickly turned green, 0.1 ml HOAc (3 M) is immediately added to the above solution. The solution was further moved into a 10 mL pressure tube, after three freeze-pump-thaw cycles, the pressure tube was heated at 120 °C for 3 days. The solid was collected by centrifugation and washed with dried THF and acetone. The yellow powder was then dried at 80 °C under vacuum for 24 h to give the Salen-COF in 51% isolated yield (76 mg, based on the total weight of TTHEPB and EDA). Anal. Cald for (C_22_H_19_NO)n: C 80.66; H 6.71; N 5.89. Found: C 79.03; H 6.39; N 5.24. To expand the scale of Salen-COF synthesis, the dosage of TTHEPB was broadened to 0.3 mmol. The obtained powder also exhibits high crystallinity (Supplementary Fig. 5). To a mixture of Salen-COF (200 mg) and Zn (OAc)_2_ (50 mg), was added dry MeOH (25 mL) under N_2_ and stirred at room temperature for 48 h. The resulting precipitate was further obtained by filtration and washed with THF, acetone to afford Zn-Salen-COF-SDU113. Moreover, we have synthesized the identified COF (Zn-Salen-COF-SDU113) on a larger scale and addressed an approach to enlarge the production of Zn-Salen-COF-SDU113. For the synthesis of 1.02 g COF, 1 g TTHEPB were dissolved in a mixture of 32 ml 1,4-dioxane and 8 ml EtOH. Then, 200 μL EDA was added, the mixed solution quickly turned green, 2 ml HOAc (3 M) is immediately added to the above solution. The solution was further moved into a 100 mL stainless autoclave and heated at 120 °C for 3 days. The solid was collected by centrifugation and Soxhlet extraction with dried 1,4-dioxane and acetone. The powder was then dried at 80 °C under vacuum for 24 h to give the Salen-COF in 85% isolated yield. The synthetic details for Zn-Salen-COF-SDU11 is similar to Zn-Salen-COF-SDU113. (see Supplementary Table 1).

### Catalytic activities evaluation

The Zn-Salen or Zn-Salen-COF-SDU113 catalysts were dried at 120 °C in vacuum for 24 h before use. A mixture of Zn-Salen or Zn-Salen-COF-SDU113 (50 mg) and n-Bu_4_NBr (600 mg, 1.8 mmol) was placed in a vacuum tube. The reaction system were refilled with CO_2_, and then 1,2-epoxypropane (25 mmol, 1.75 mI) was added to the tube by a syringe. After being stirred for 48 h at 25 °C, the mixture was then dissolved with ethyl acetate and the insoluble solid was collected by centrifuge and washed by fresh CH_2_Cl_2_ and dried by vacuum for cyclic utilization. After reduced pressure distillation, the filtrate generated a pale yellow oily substance. Further purification of the crude product was carried out through column chromatography. The propylene carbonate and 2,3-epoxybutane were confirmed by gas chromatography (Supplementary Figs. 9 and 10).

## Supplementary information

Supplementary Information

Peer Review File

## Data Availability

The authors declare that all the other data supporting the findings of this study are available within the Article and its Supplementary Information files and from the corresponding author upon request. 10994 optimized metal/Salen-COF structures are provided at 10.6084/m9.figshare.12488519. Source data are provided with this paper.

## Source data

Source Data
