# Supplementary material for: Enhanced carbon dioxide conversion at ambient conditions via a pore enrichment effect
Source: Nat Commun. 2020 Sep 8;11:4481. doi: 10.1038/s41467-020-18154-9 (PMC7479596; doi:10.1038/s41467-020-18154-9)
Supplement: Supplementary file 1 — Supplementary Information [file 41467_2020_18154_MOESM1_ESM.pdf]

Enhanced carbon dioxide conversion at ambient conditions via pore enrichment effect

Wei Zhou[1,#], Qi-Wen Deng[1,4,#], Guo-Qing Ren[1], Lei Sun[1,], Li Yang[2,3], Yi-Meng Li[1], Dong Zhai[1], Yi-Hong Zhou[4], Wei-Qiao Deng[1,2,*]*

[1] *Institute of Molecular Sciences and Engineering, Institute of Frontier and Interdisciplinary Science, Shandong University, Qingdao 266237, China.*

[2] *State Key Laboratory of Molecular Reaction Dynamics, Dalian National Laboratory for Clean Energy, Dalian Institute of Chemical Physics, Chinese Academy of Sciences, Dalian 116023, China.*

[3] *University of the Chinese Academy of Sciences, Beijing 100039, China.*

[4] *Collage of Hydraulic & Environmental Engineering, China Three Gorges University, Yichang, Hubei 443002, P. R. China.*

* Correspondence and requests for materials should be addressed to L. S.: slei@sdu.edu.cn and W.-Q. D.: dengwq@sdu.edu.cn).

Supplementary Methods

M/Salen unite structures:

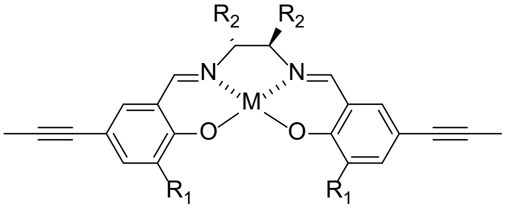

R_1: H, CH_3, OCH_3, $C(CH_3)_3$

R_2: CH_2-CH_2, CH_2-$(CH_2)_2$-CH_2

Linker group libraries:

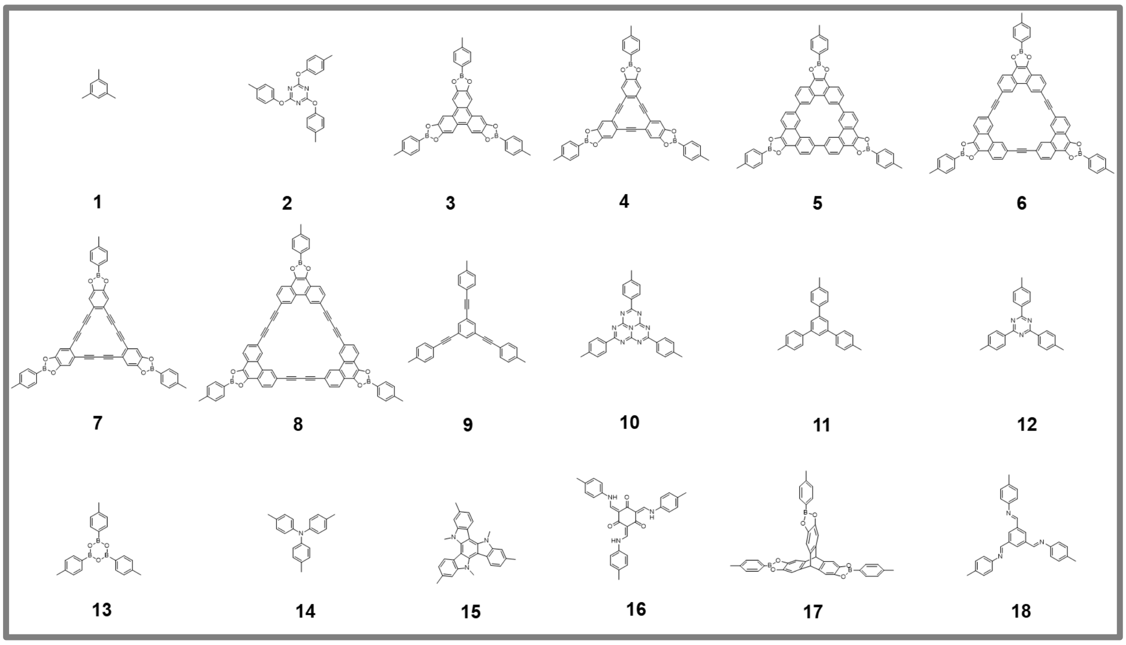

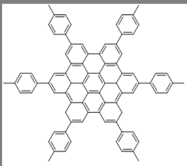
19
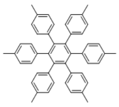
20
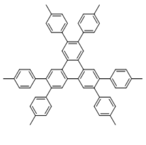
21
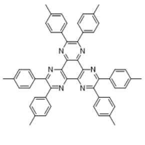
22
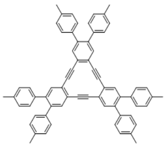
23
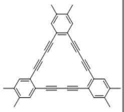
24
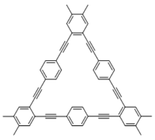
25

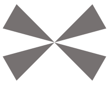

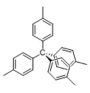
26
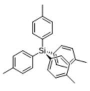
27
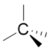
28
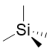
29
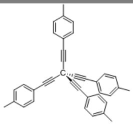
30
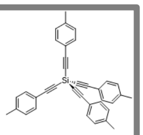
31
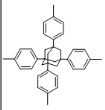
32
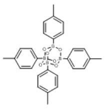
33

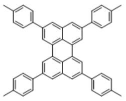
34
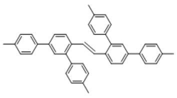
35
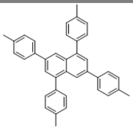
36
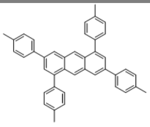
37
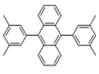
38
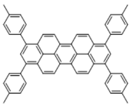
39
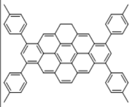
40
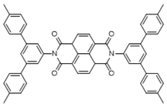
41
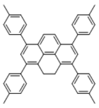
42
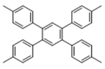
43
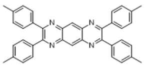
44
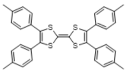
45
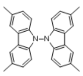
46
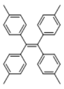
47
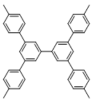
48
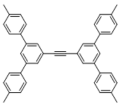
49
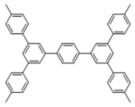
50
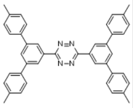
51

Supplementary Methods.

Chemicals. All solvent were purchased from commercial sources. Tetrahydrofuran, acetone, methanol, ethanol, and acid were bought from Sinopharm Chemical Reagent Co., Ltd. Tetrahydrofuran, ethanol were dried before used. Ethylenediamine (EDA) was purchased from Macklin Chemical Reagent. Dimethylacetamide (DMAc) and aqueous acetic were bought from Aladdin Reagent. 5,5'-((5'-(4-((3-(tert-butyl)-5-formyl-4-methylphenyl)ethynyl)phenyl)-[1,1':3',1''-terphenyl]-4,4''-diyl)bis(ethyne-2,1-diyl))bis(3-(tert-butyl)-2-hydroxybenzaldehyde) (TTHEPB, purity: 97 %) was bought from Jilin Chinese Academy of Sciences-Yanshen Technology Co., Ltd.

Physical characterizations. The ^{13}C Cross Polarization-Magic Angle Spinning (CP-MAS) NMR spectra of Salen COF was conducted on a Varian Infinity-400 spectrometer. The Brunauer-Emmett-Teller (BET) surface areas of Salen COF was measured at 77 K by using a Quantachrome Automated Surface Area & Pore Size Analyzer. Pore size distributions was estimated by nonlocal density functional theory (NLDFT). The following CO_2 sorption isotherms for Zn/Salen-COF at 298 K was measured by Micromeritics ASAP 2050 system volumetric adsorption analyzer. The powder X-ray diffraction (PXRD) pattern was recorded on a Cu-Kα X-ray radiation source (λ=0.154056 nm) incident radiation by a Rigaku MiniFlEX 600 instrument over the range of 2θ =2.0~40.0° with a step size of 0.02° per step. The FT-IR spectra were recorded by Thermo Nicolet iS50 in the range from 400 to 4000 cm^{-1}. Morphological information for Zn Salen-COF were obtained from field-emission scanning electron microscope (FE-SEM, JEOLJSM 6700F) and transmission electron microscopy (TEM, JEOLJEM-2010F). The X-ray photoelectron spectroscopic (XPS) was obtained from an ESCALAB 250 with a monochromatic Al Kα X-ray source. Thermogravimetric analyses (TGA) was recorded on a Netzsch Model STA 449C microanalyzer heated from 25 °C to 900 ^{o}C in nitrogen atmosphere.

Supplementary Table 1. Simulated results and synthesis conditions for Zn-Salen-COF-SDU (11 and 113).

Entry	Simulated Results	Ligands	Solvents &Temp.	Structure
1	P_{in} :25.3 bar<br>I_k: 29.3	TTHEPB / EDA<br>0.055 mmol / 0.45 mmol<br>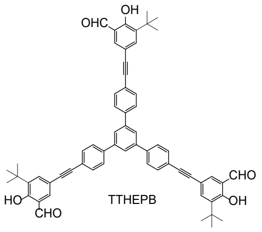	6.4 ml THF<br>1.6 ml EtOH<br>0.1 ml (3 M)<br>HOAc<br>120 °C, 3 days	Salen-COF-113<br>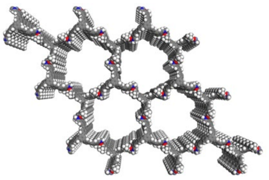
2	Pin :6.0 bar<br>I_k: 6.2	THEPB<br>0.3 mmol / 0.45 mmol<br>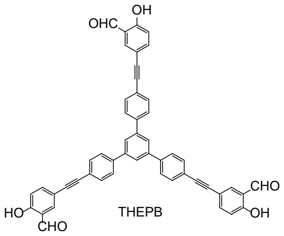	3.2 ml 1,4-<br>Dioxane<br>0.8 ml EtOH<br>0.2 ml (3 M)<br>HOAc<br>120 °C, 3 days	Salen-COF-11<br>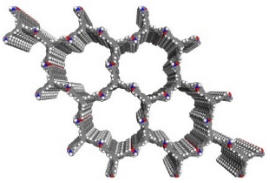

* P_{in} is the internal pressures in the sorption simulations. I_k is the increment of reaction rate under the pore enrichment effect.

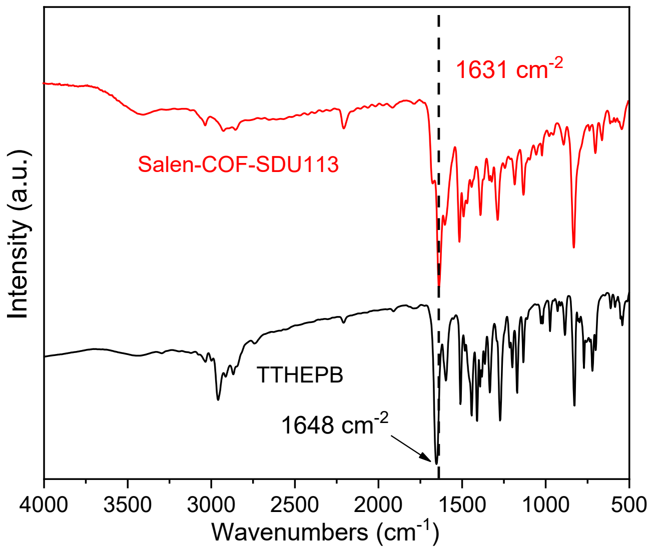

Supplementary Figure 1. FT-IR spectra for TTHEPB ligands and Salen-COF-SDU113.

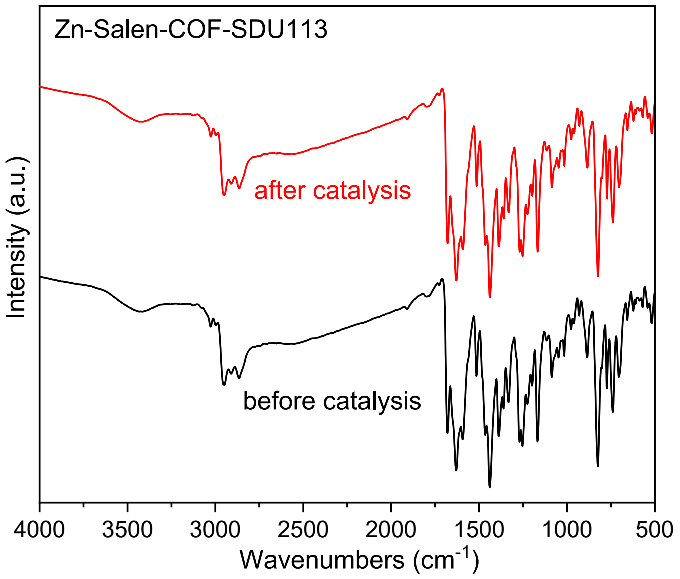

Supplementary Figure 2. FT-IR spectra for Zn-Salen-COF-SDU113 before and after catalysis.

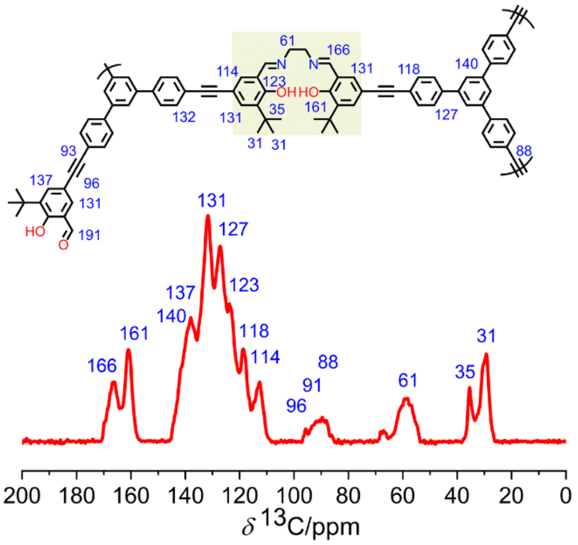

Supplementary Figure 3. The ^{13}C CP/MAS NMR spectrum of Salen-COF-113.

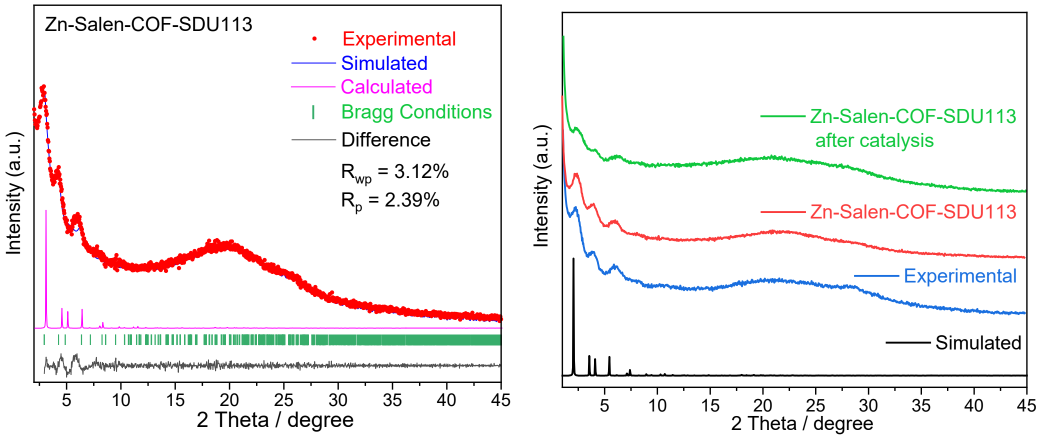

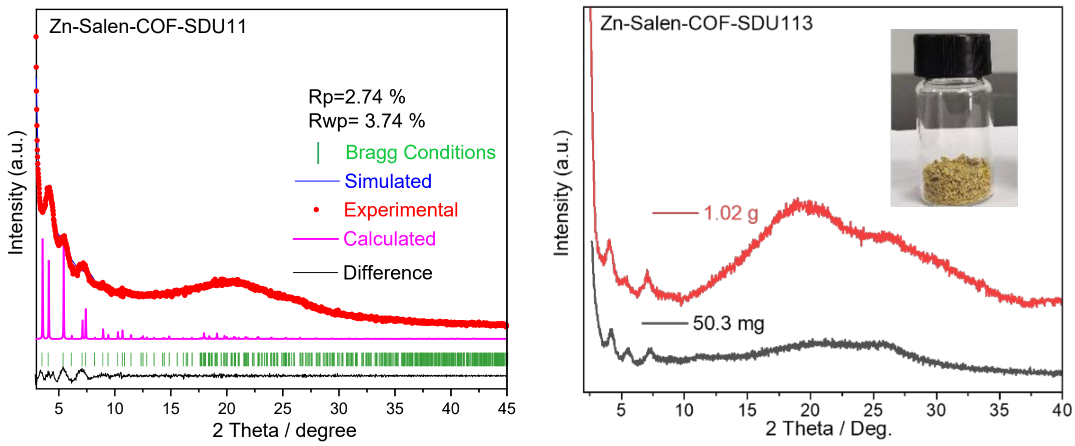

Supplementary Figure 4. PXRD patterns of simulated, experimental Zn-Salen-COF-SDU113 (before and after catalysis undergoing 5 cycles), Zn-Salen-COF-SDU11 and Zn-Salen-COF-SDU113 synthesized by different dosage.

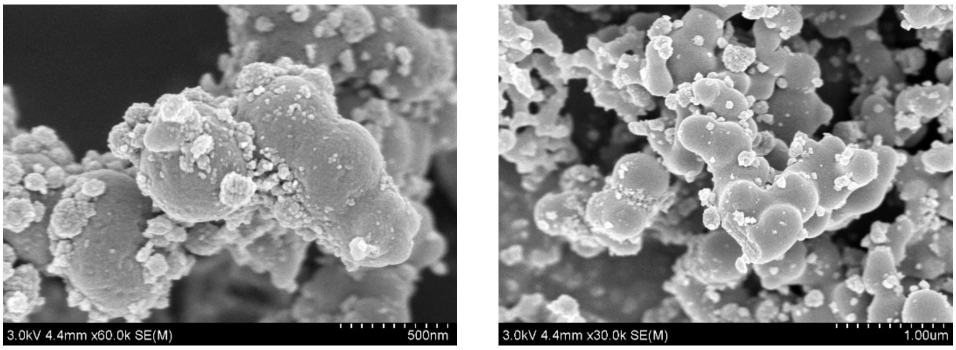

Supplementary Figure 5. SEM images of Zn-Salen-COF-SDU113.

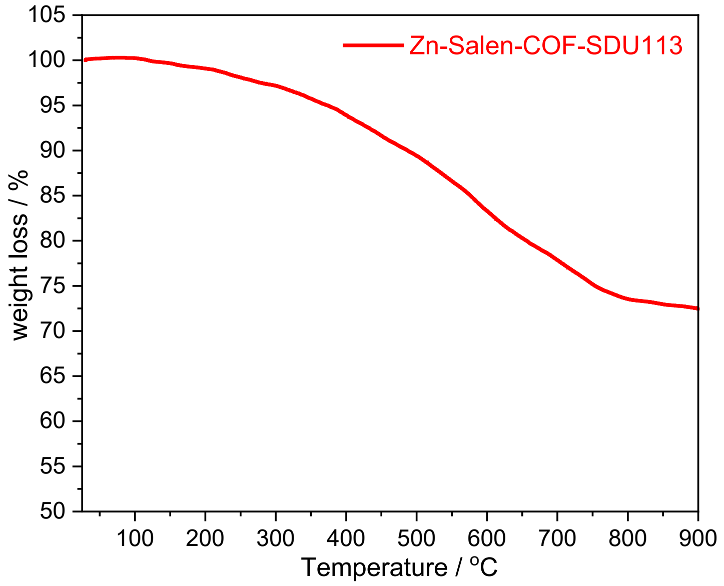

Supplementary Figure 6. TG analysis for Zn-Salen-COF-SDU113.

Supplementary Table 2. Fractional atomic coordinates for the unit cell of Zn-Salen-COF-SDU113.

Space group: ***P*321**<br>a = b = 43.887 Å, c = 8.418 Å<br>*α* = *β* = 90º, *γ* = 120º								
Atom	x (Å)	y (Å)	z (Å)		Atom	x (Å)	y (Å)	z (Å)
H1	0.62254	0.60618	0.66728		C8	0.59864	0.54772	0.31693
C2	0.55441	0.46335	0.82331		N9	0.58991	0.56947	0.37532
H3	0.61472	0.64131	0.60717		C10	0.60674	0.61321	0.58793
C4	0.49426	0.44647	0.71854		C11	0.54392	0.45857	0.52692
O5	0.54907	0.51516	0.59087		C12	0.54793	0.43728	0.41231
C6	0.55668	0.49465	0.48833		C13	0.56524	0.45121	0.26920
C7	0.57691	0.50927	0.34697		C14	0.58035	0.48699	0.23842
H15	0.62285	0.55757	0.24997		H16	0.53864	0.40985	0.43444
H17	0.59517	0.49735	0.12897		C18	0.57054	0.42923	0.15947
C19	0.57646	0.41142	0.07022		C20	0.58602	0.39110	-0.02989
C21	0.58107	0.35896	0.02537		C22	0.60493	0.40574	-0.16987
C23	0.59710	0.34260	-0.05246		H24	0.56662	0.34777	0.13442
C25	0.62137	0.38954	-0.24770		H26	0.60810	0.43060	-0.21299
C27	0.61859	0.35839	-0.18618		H28	0.59498	0.31906	-0.00026
H29	0.63755	0.40219	-0.35090		C30	0.64251	0.34564	-0.23964
C31	0.67886	0.36948	-0.24380		C32	0.52730	0.44341	0.69173
C33	0.51612	0.40443	0.71407		H34	0.68832	0.39739	-0.23126
H35	0.56624	0.49229	0.81047		H36	0.54142	0.45647	0.94143
H37	0.57584	0.45640	0.82243		H38	0.47654	0.43604	0.61443
H39	0.50113	0.47414	0.73733		H40	0.47991	0.43194	0.82661
H41	0.53998	0.40189	0.70319		H42	0.49551	0.38723	0.62869
H43	0.50680	0.39611	0.83721		Zn259	0.55356	0.55356	0.50000

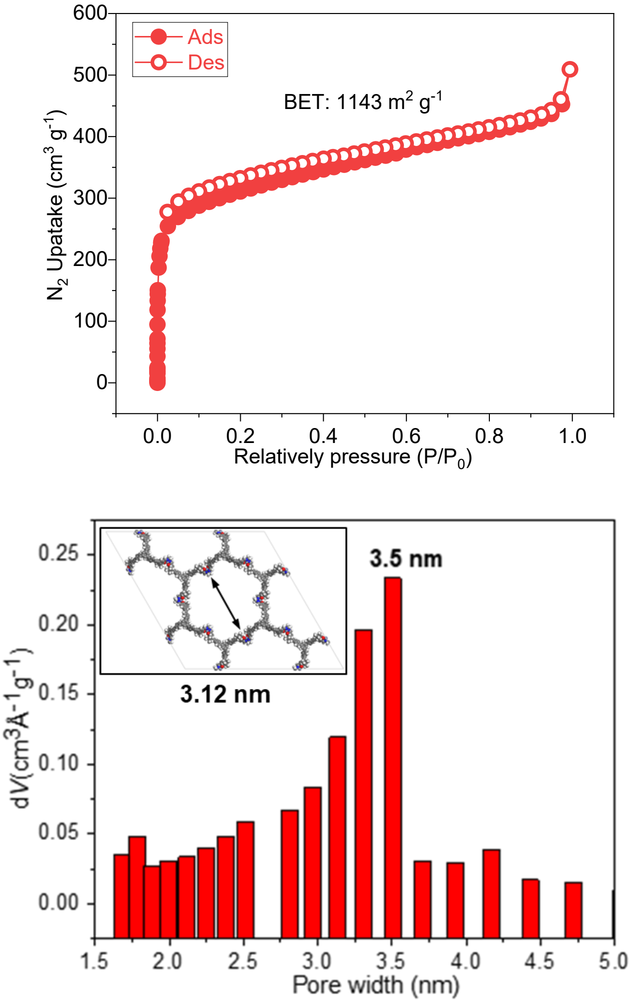

Supplementary Figure 7. The N_2 adsorption-desorption isotherms (77 K) and corresponding pore size distribution for Zn-Salen-COF-SDU113.

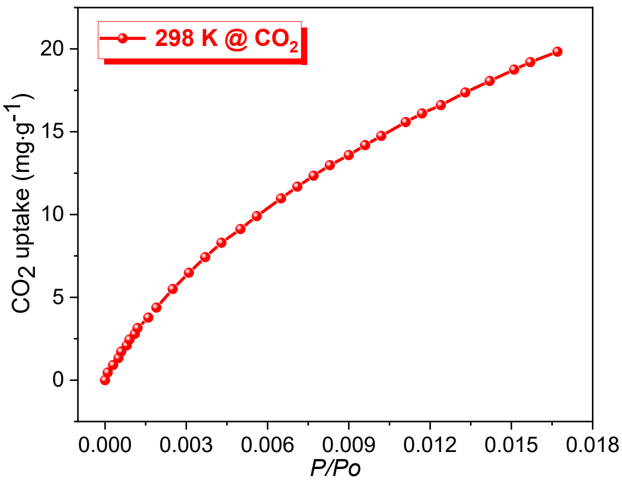

Supplementary Figure 8. CO_2 adsorption isotherms measured at 298 K for Zn-Salen-COF-SDU113.

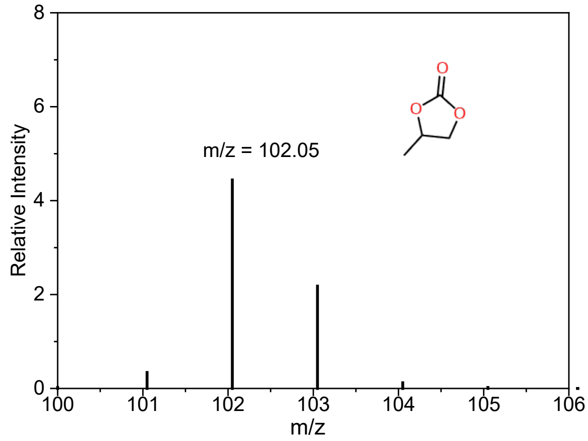

Supplementary Figure 9. Mass spectra analysis for the obtained propylene carbonate.

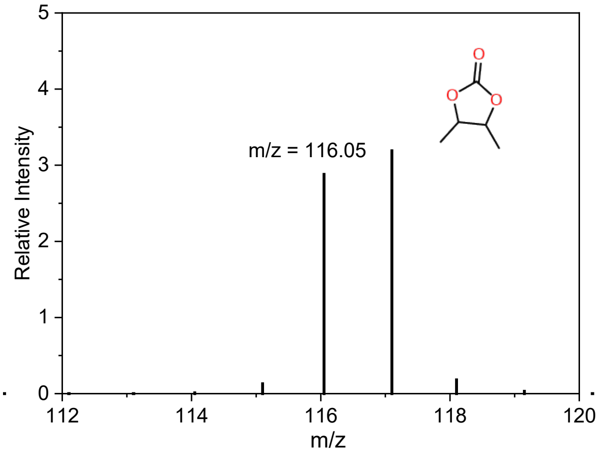

Supplementary Figure 10. Mass spectra analysis for the obtained carbonic acid 2, 3-butanediyl.

Supplementary Table 3. Comparison of cyclic carbonate yields of different porous crystalline materials.

Entry	Catalysts	TBAB (mmol)	Time (h)	Yield (%)	TOF	TON	Ref
1	Zn-Salen-COF-SDU11	1.8 mmol	48	84.2	5.95	285.7#	This work
2	Zn-Salen-COF-SDU113	1.8 mmol	48	97.3	63.35	3040.6¶	
3	Zn-Salen-COF-SDU113	1.95 mmol	48	98.2	63.94¶	3068.9¶	
4	Zn-Salen-COF-SDU113	1.8 mmol	48	97.3	15.84	760.2#	
5	Zn-Salen-COF-SDU113	1.95 mmol	48	98.2	15.99	767.4#	
6	MOF-505	1.74 mmol	48	48.0	4	192#	Ref. 1
7	MMCF-2	1.74 mmol	48	95.4	5.3	254.4#	
8	Cu(tactmb)	1.74 mmol	48	47.5	7.92	380#	
9	HKUST-1	1.74 mmol	48	49.2	4.1	196.8#	
10	HKUST-1	1.95 mmol	36	65	135.4¶	4874.4¶	Ref. 2
11	Cu-MOF 1	1.95 mmol	48	96	200¶	9600¶	
12	HKUST-1	1.95 mmol	36	30.0	15.5¶	558¶	Ref. 3
13	CASFZU-1	1.95 mmol	36	98.0	54¶	1944¶	

¶ TON results based on per metal active sites (paddlewheel Cu_2 cluster or per Zn active sites);

\# TON = (moles of product) / (moles of metal in the catalyst);

Reaction conditions: Temperature: 25 °C; CO_2 pressure: 0.1 MPa.

Supplementary References

1. Gao, W. Y., et al. Crystal Engineering of an nbo Topology Metal-Organic Framework for Chemical Fixation of CO_2 under Ambient Conditions. *Angew. Chem. Int. Ed.* **53**, 2615-2619 (2014).
2. Li, P. Z., et al. A Triazole-Containing Metal–Organic Framework as a Highly Effective and Substrate Size-Dependent Catalyst for CO_2 Conversion. *J. Am. Chem. Soc.* **138**, 2142-2145 (2016).
3. Yu, Z., et al. Unconventional CN vacancies suppress iron-leaching in Prussian blue

analogue pre-catalyst for boosted oxygen evolution catalysis. *Nat. Commun.* **10**, 2799 (2019).
